# Supplementary material for: Assessment of the human placental microbiome in early pregnancy
Source: Front Med (Lausanne). 2023 Jan 19;10:1096262. doi: 10.3389/fmed.2023.1096262 (PMC9892641; doi:10.3389/fmed.2023.1096262)
Supplement: Supplementary file 1 [file Data_Sheet_1.PDF]

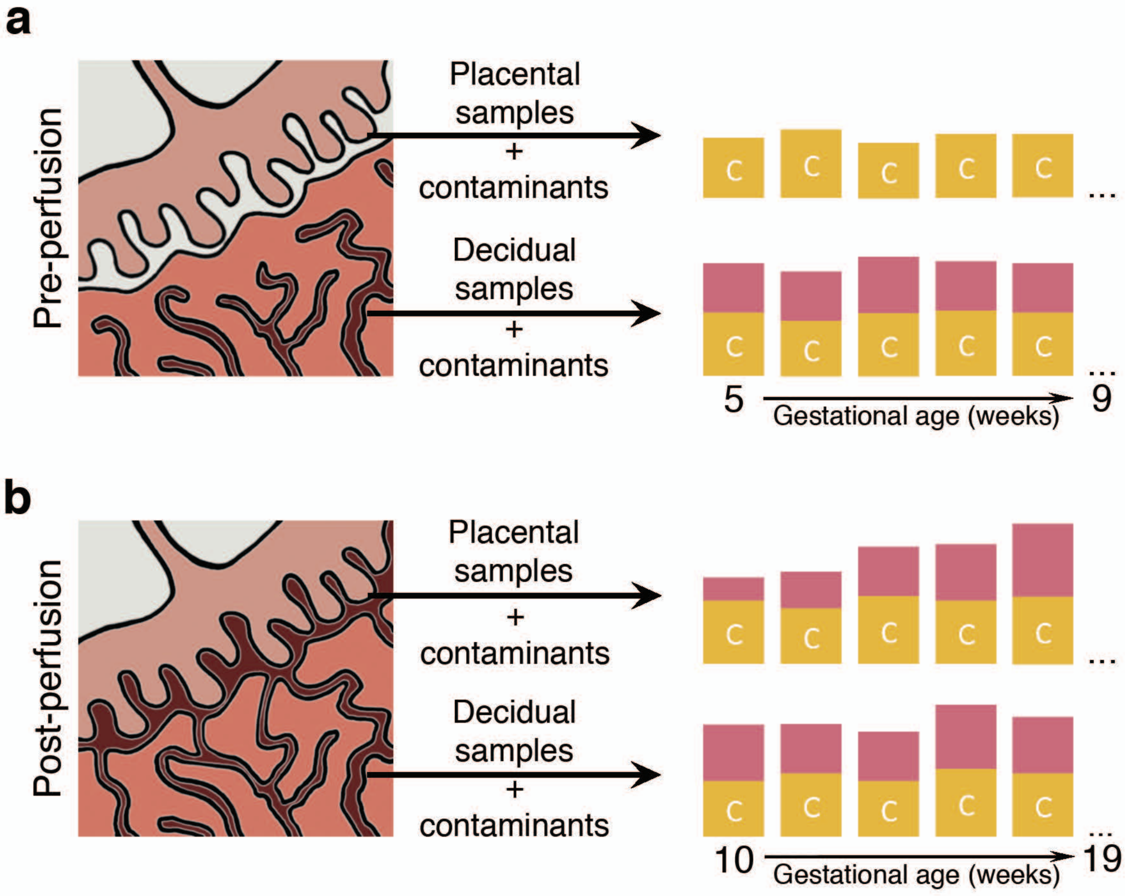

Supplemental Figure S1. The microbiome component of this study is designed to detect an increase in placental bacterial DNA compared to decidua above background bacterial contamination. During the anoxic phase of embryonic development the placenta is not perfused by the maternal circulation and not exposed to blood-borne bacteria. During this pre-perfusion phase,only bacterial contaminants introduced during delivery sampling (yellow boxes, labeled "C") should be detectable in placental samples while decidual samples contain bacteria derived from the maternal circulation (red boxes) as well as contaminants (a). After onset of perfusion the placenta is exposed to blood-borne bacteria that should become detectable as increasing bacterial abundance and/or diversity over time compared to decidua (b).

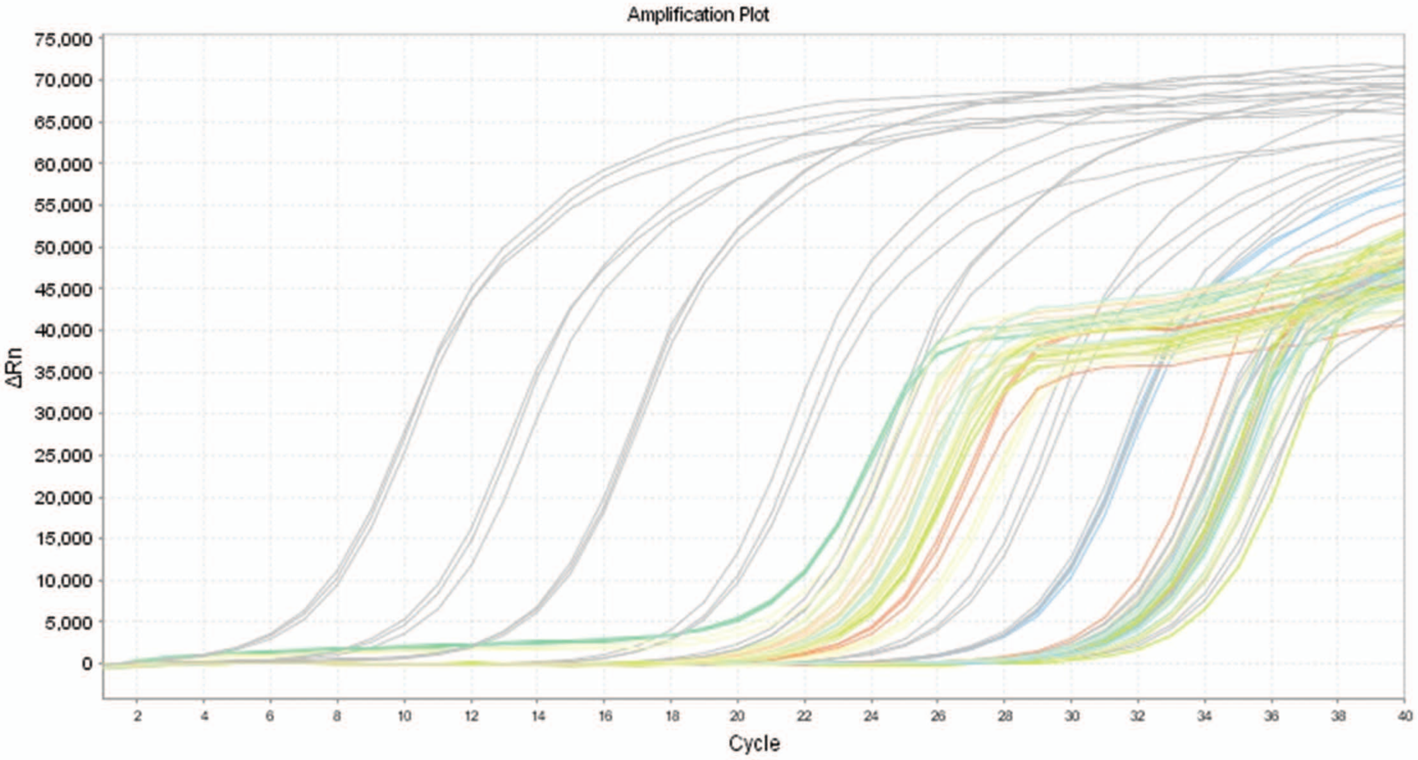

Supplemental Figure S2. Quantitative 16S PCR amplifications curves.The left cluster of colored curves represent the tissue samples and the right cluster of colored curves represent the PBS wash samples. The grey curves represent 10 fold dilutions of the bacterial 16S rRNA control.

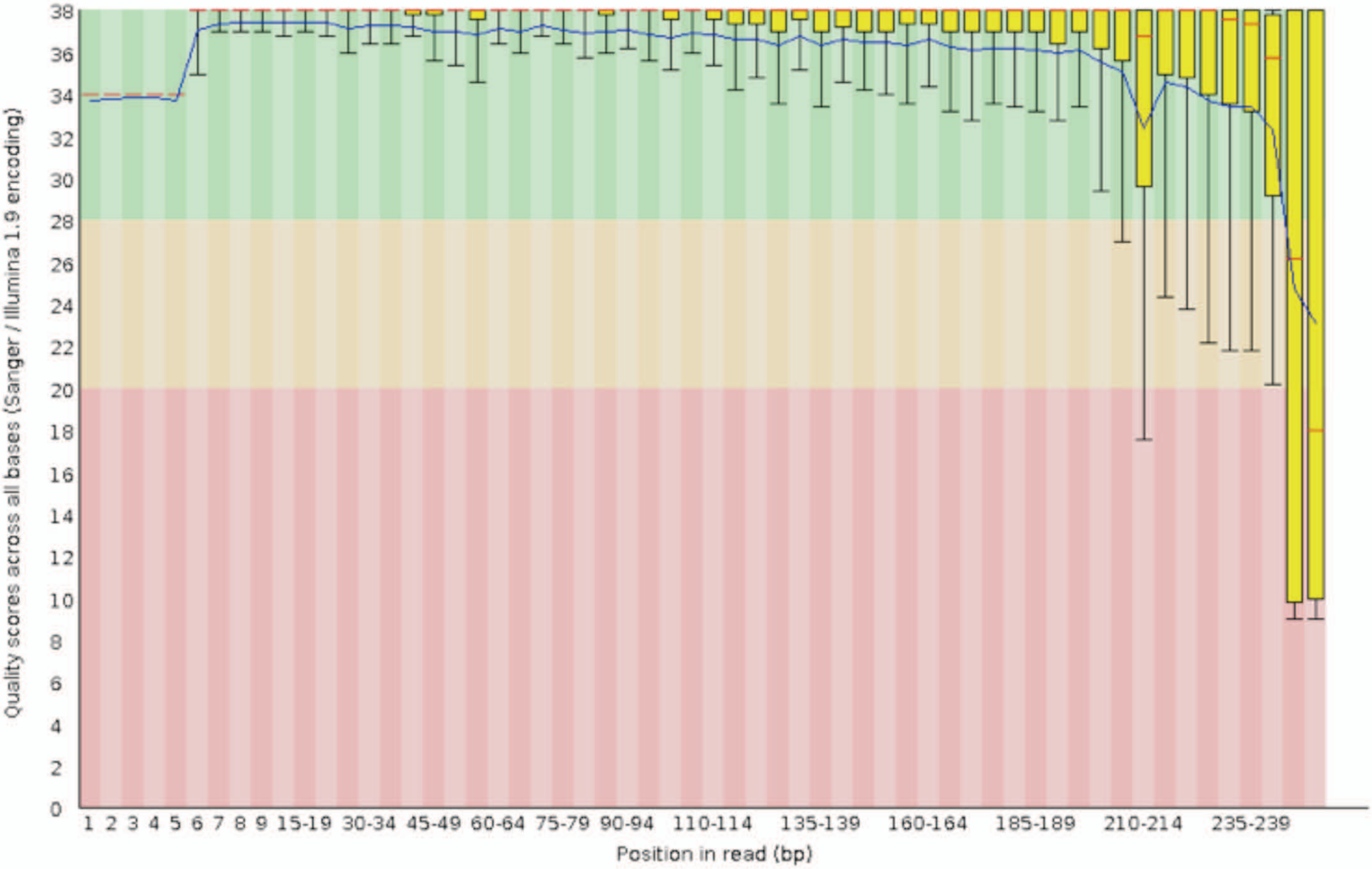

Supplemental Figure S3. Per-base raw Phred33 scores of the forward sequencing reads. Scores are determined using FastQC 0.11.5 and assess the accuracy of sequencing and indicates the probability that a particular base has been called incorrectly Green (Q score 28 or greater, probability of an incorrect call 1/933 or less) indicates high quality, yellow reasonable, and red poor. The blue line indicates mean quality, the red line indicates median value, yellow box the 25-75th percentile, and the whiskers the 10-90th percentile.

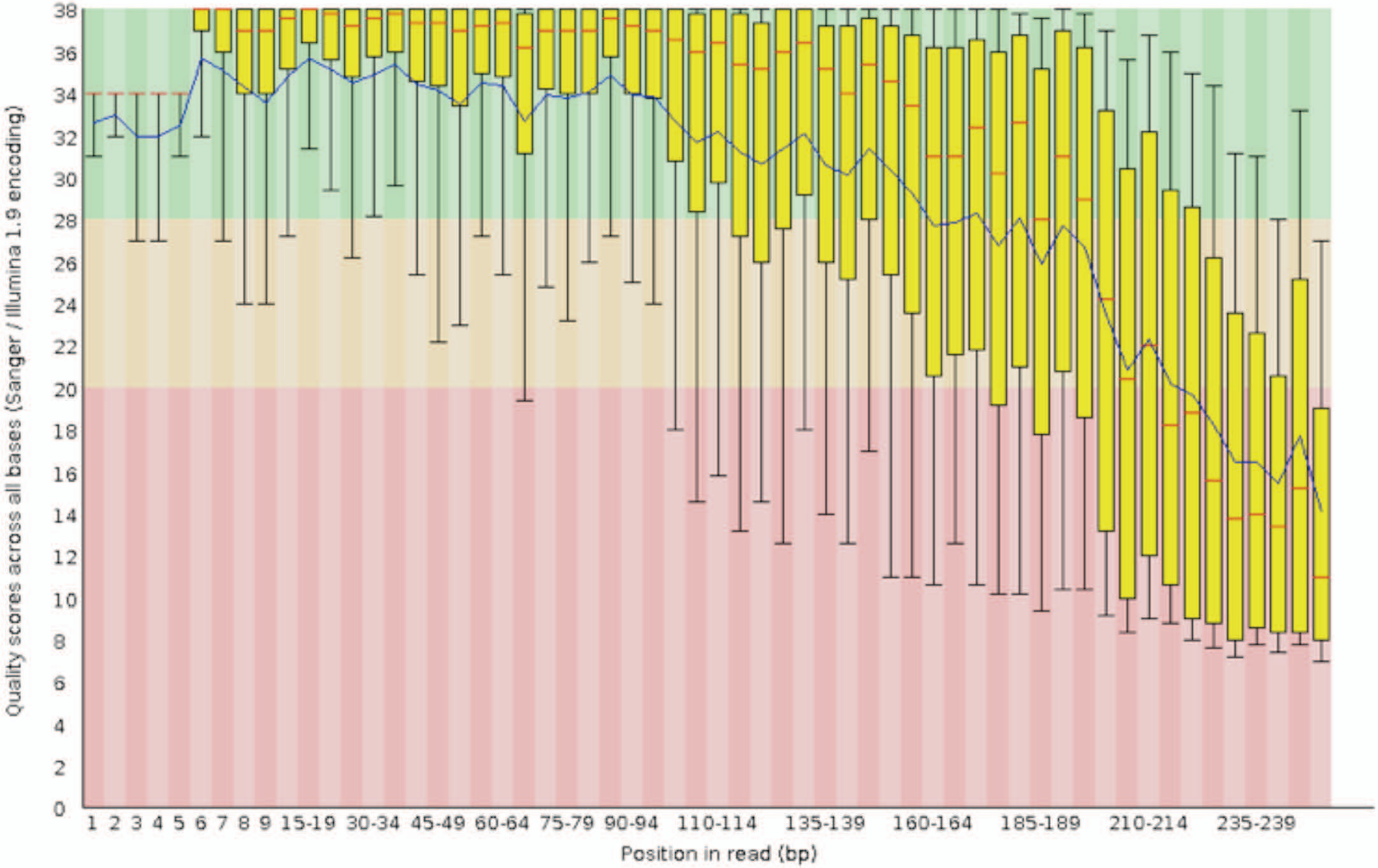

Supplemental Figure S4. Per-base raw Phred33 scores of the reverse sequencing reads. Scores are determined using FastQC 0.11.5 and assess the accuracy of sequencing and indicates the probability that a particular base has been called incorrectly. Green (Q score 28 or greater, probability of an incorrect call 1/933 or less) indicates high quality, yellow reasonable, and red poor. The blue line indicates mean quality, the red line indicates median value, yellow box the 25-75th percentile, and the whiskers the 10-90th percentile.

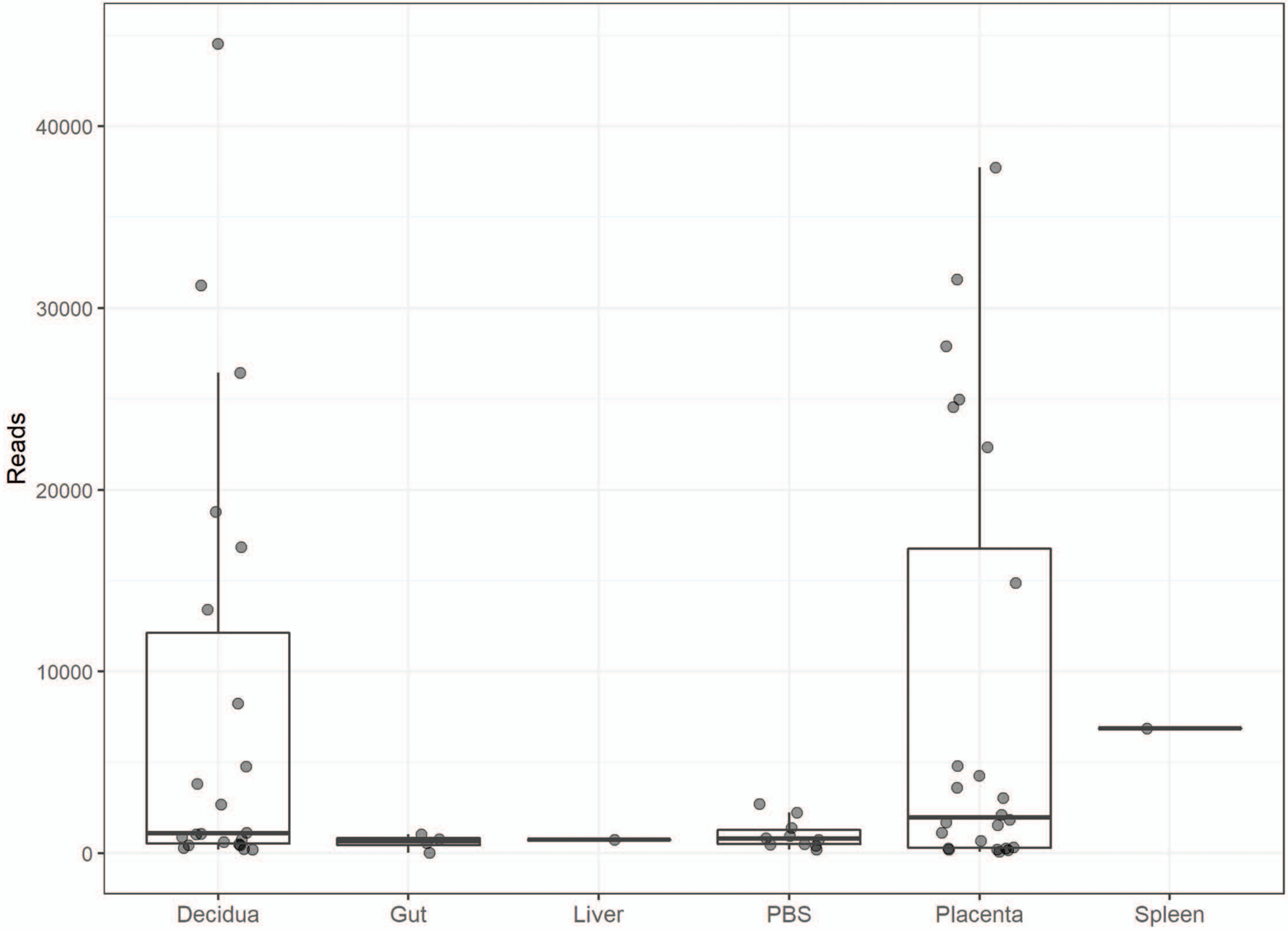

Supplemental Figure S5. Total number of quality filtered reads per sample. Reads include high-quality sequences that align with 16Sv4, are clustered into OTUs, and are assigned taxonomic classification. Ambiguous or low-quality data are excluded from subsequent analyses.

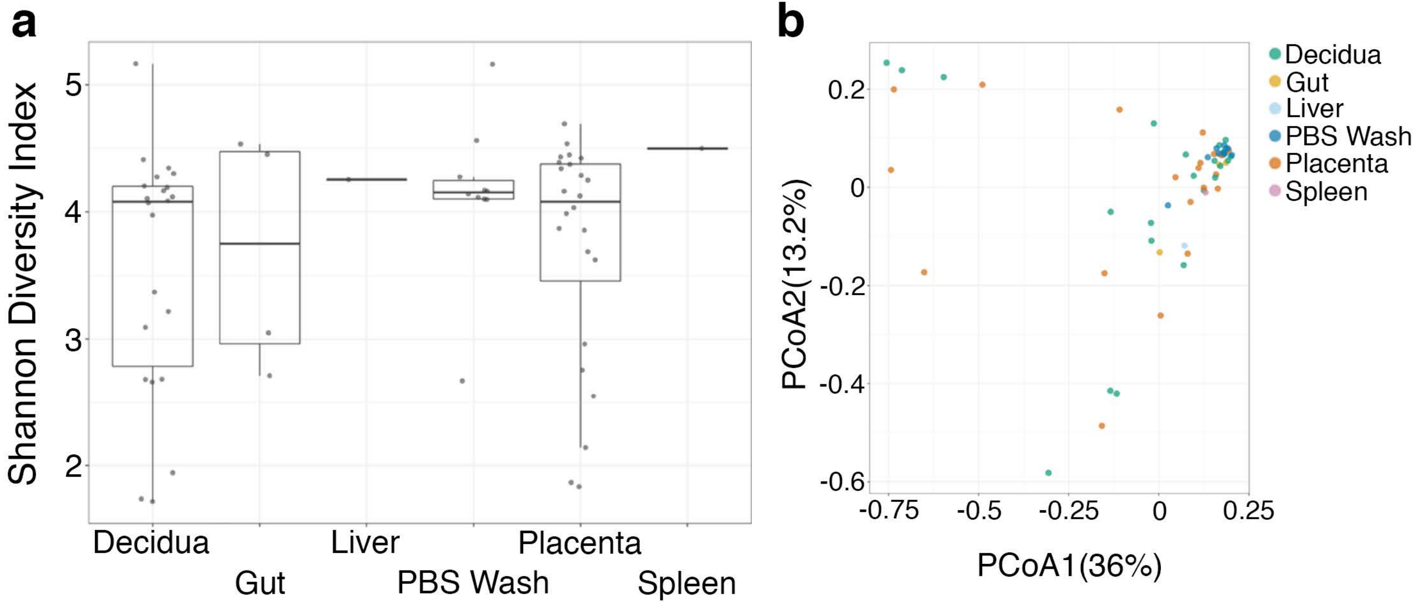

Supplemental Figure S6. Bacterial diversity between samples. Average diversity is similar between maternal decidua, placenta, fetal tissue, and phosphate buffered saline (PBS) wash samples (a). Principal coordinate analysis demonstrating similarity between samples (b). Box plots encompass the mean, upper and lower quartiles.

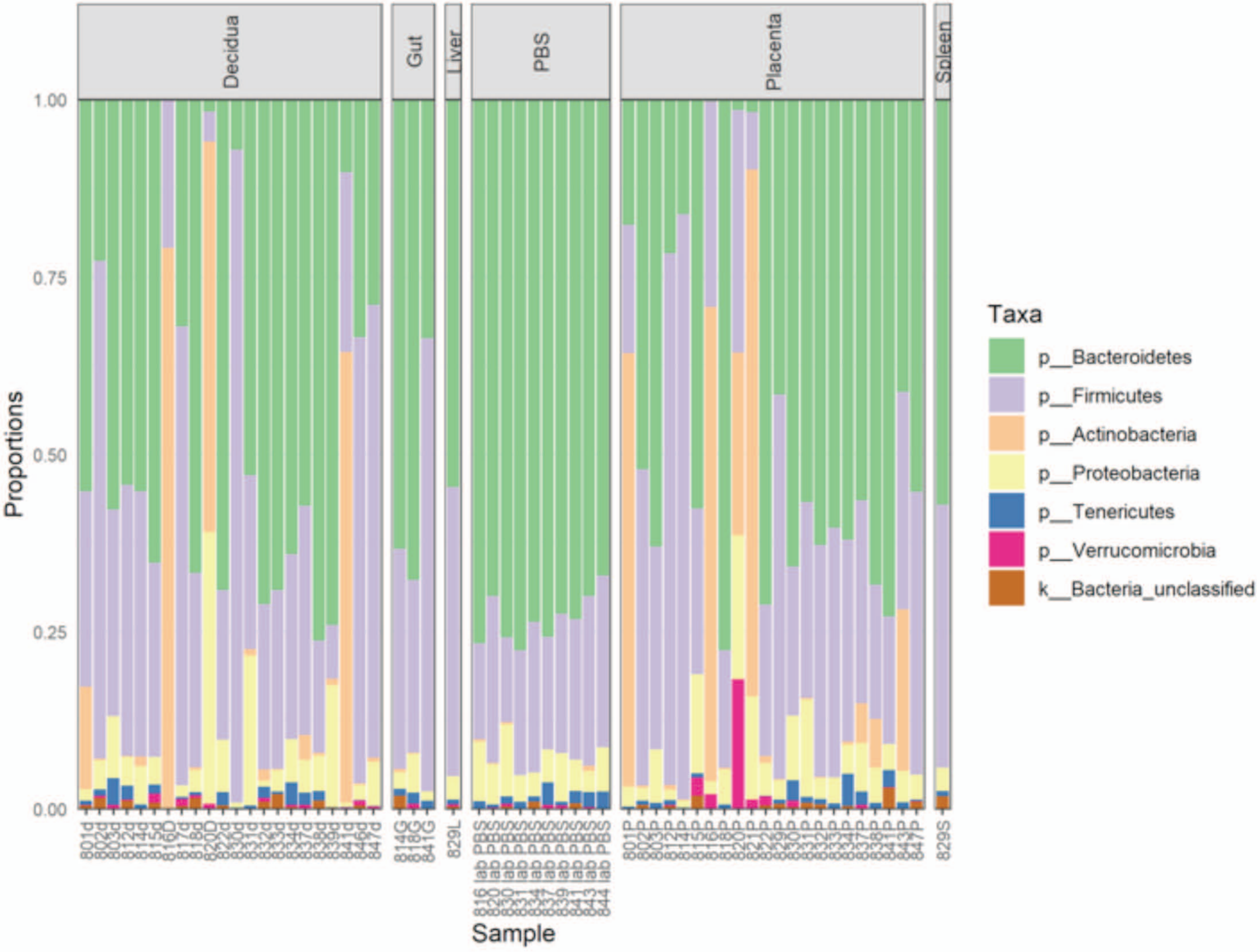

Supplemental Figure S7. Taxonomic distribution by phylum. Decidua, placenta, and fetal tissues and phosphate buffered saline (PBS) wash samples. Unfilled portion of the bar plots represent lower abundance taxa.

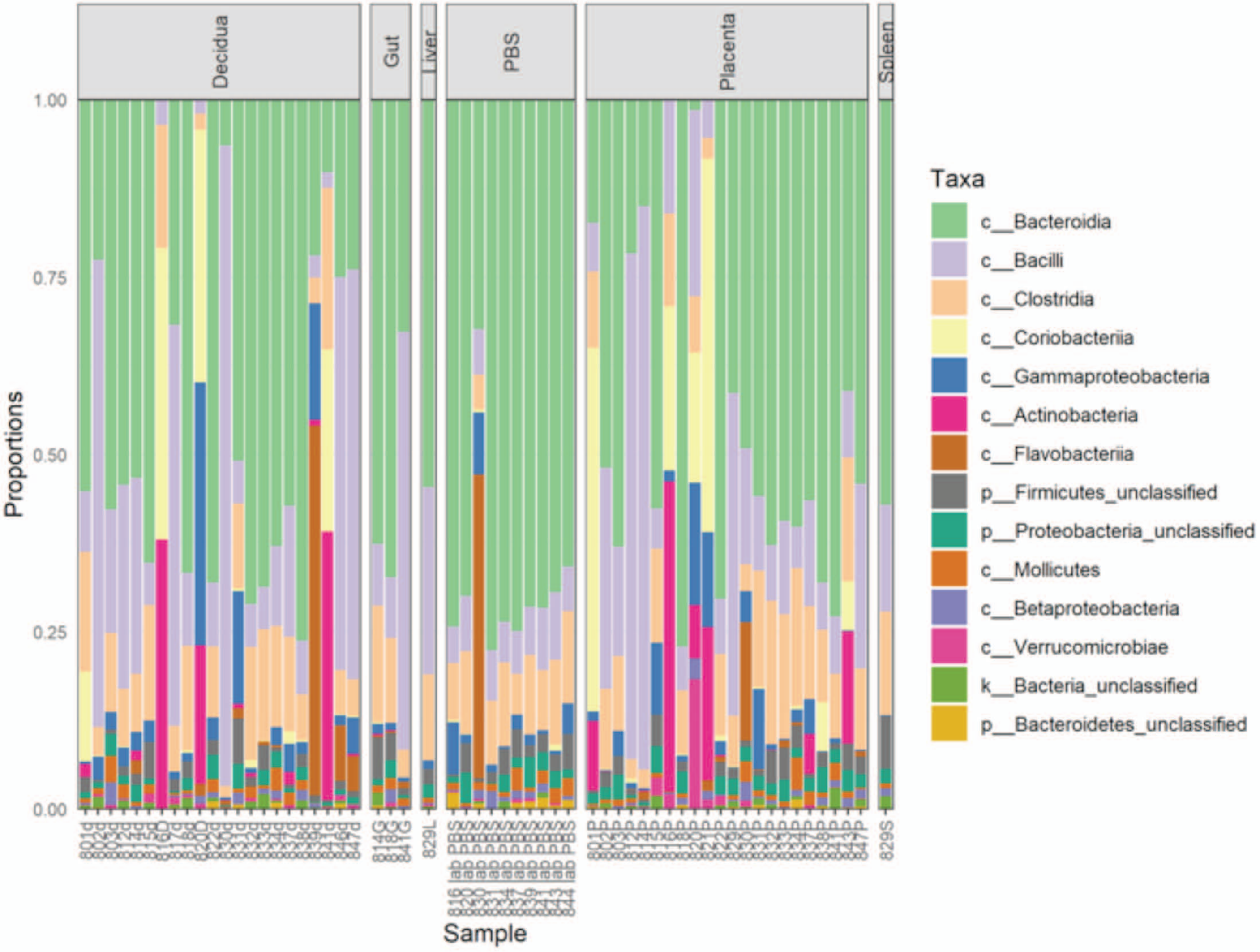

Supplemental Figure S8. Taxonomic distribution by class. Decidua, placenta, and fetal tissues and phosphate buffered saline (PBS) wash samples.

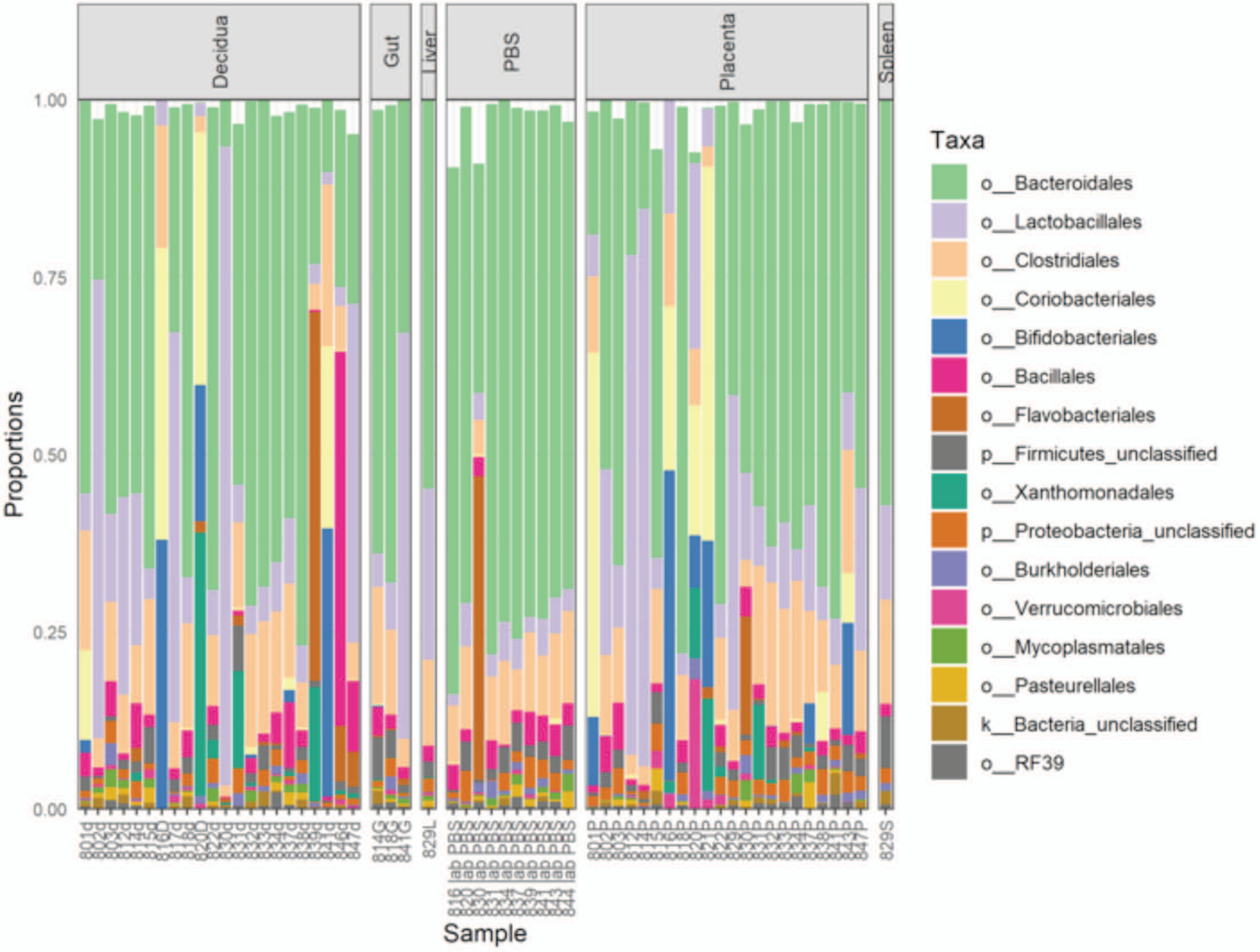

Supplemental Figure S9. Taxonomic distribution by order. Decidua, placenta, and fetal tissues and phosphate buffered saline (PBS) wash samples. Unfilled portion of the bar plots represent lower abundance taxa.

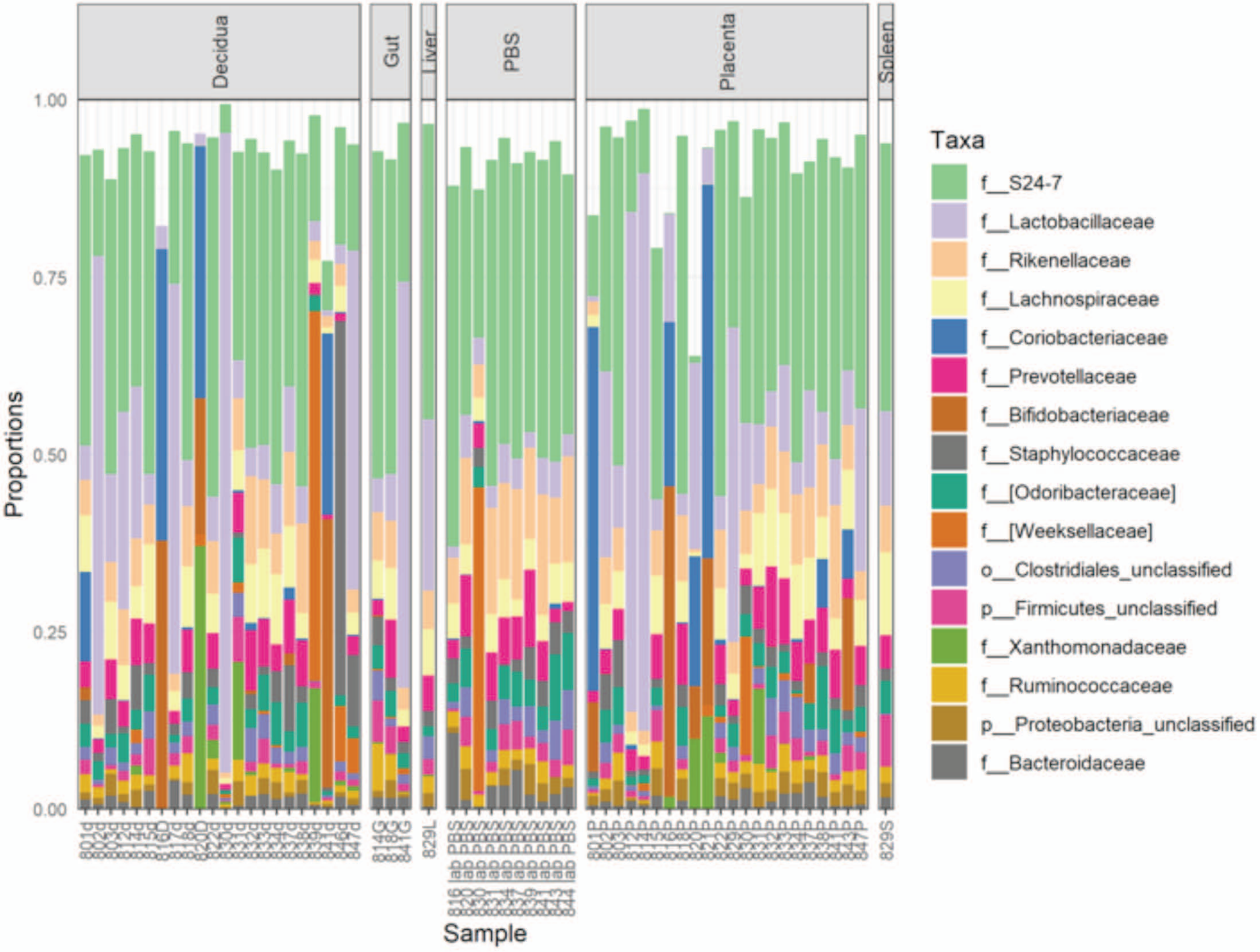

Supplementarl Figure S10. Taxonomic distribution by family. Decidua, placenta, and fetal tissues and phosphate buffered saline (PBS) wash samples. Unfilled portion of the bar plots represent lower abundance taxa.

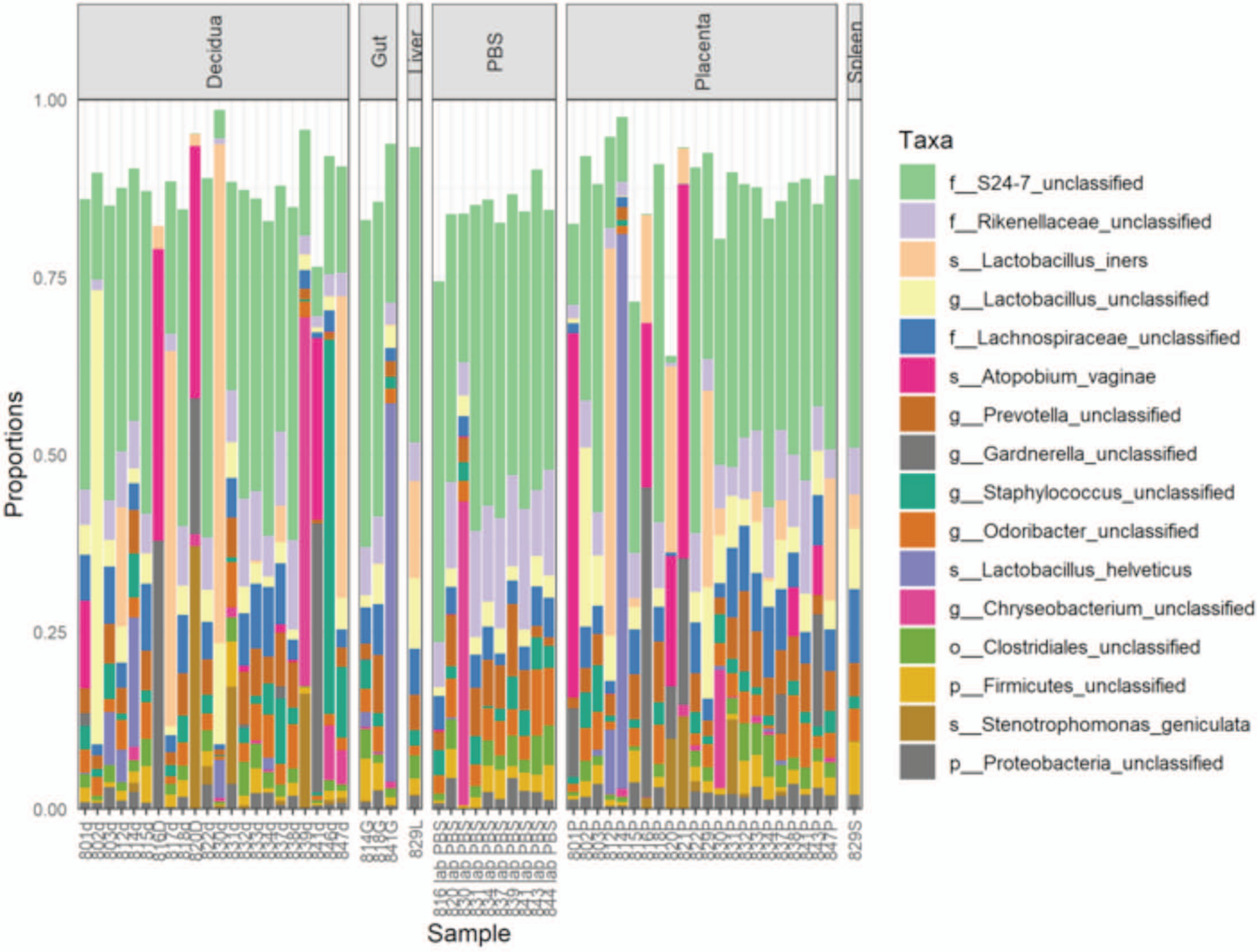

Supplemental Figure S11. Taxonomic distribution by genus/species. Decidua, placenta, and fetal tissues and phosphate buffered saline (PBS) wash samples. Unfilled portion of the bar plots represent lower abundance taxa.

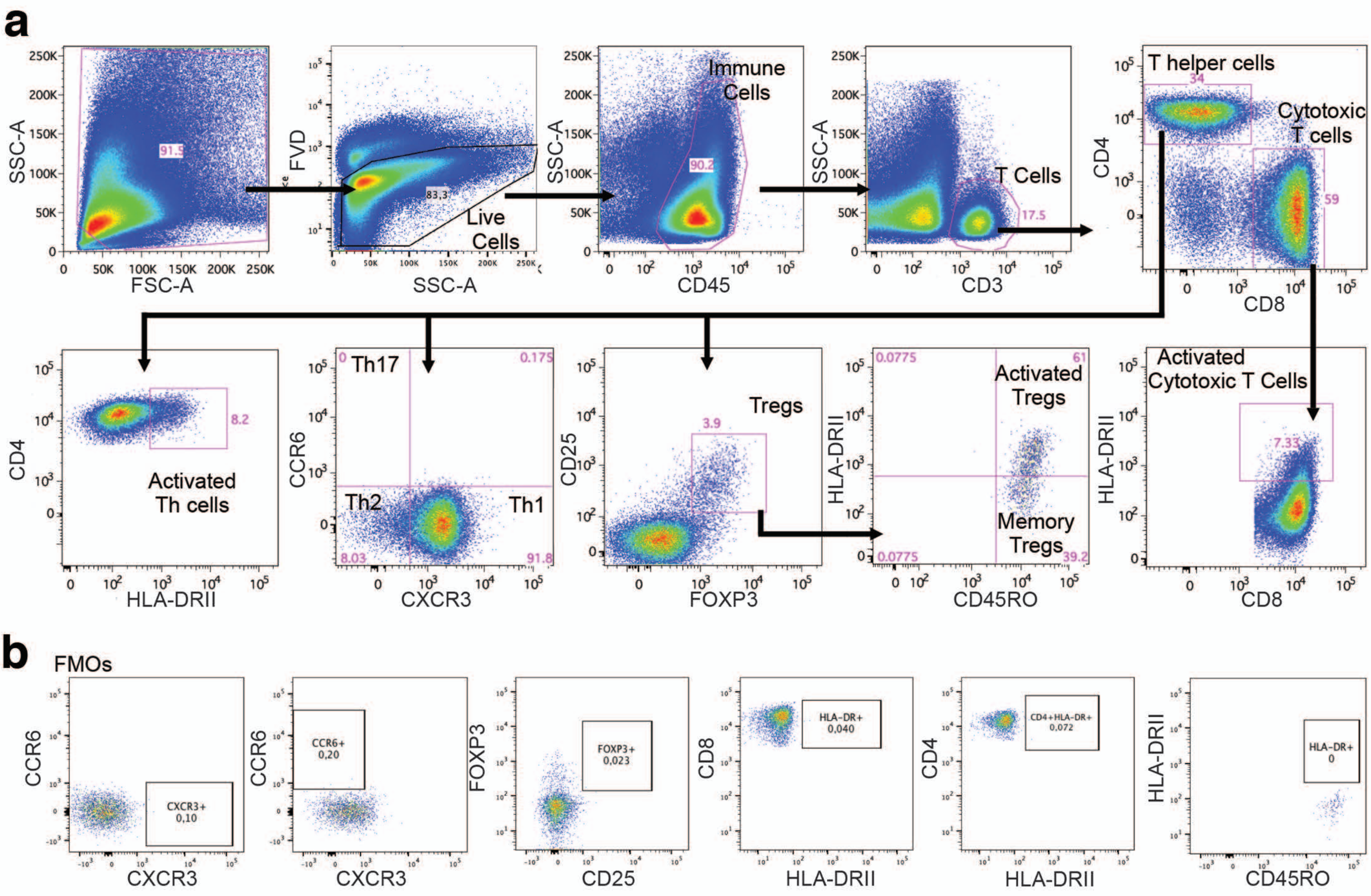

Supplemental Figure S12. Flow cytometry gating strategies. T cell subsets (a) and fluorescence minus one (FMO) controls (b).

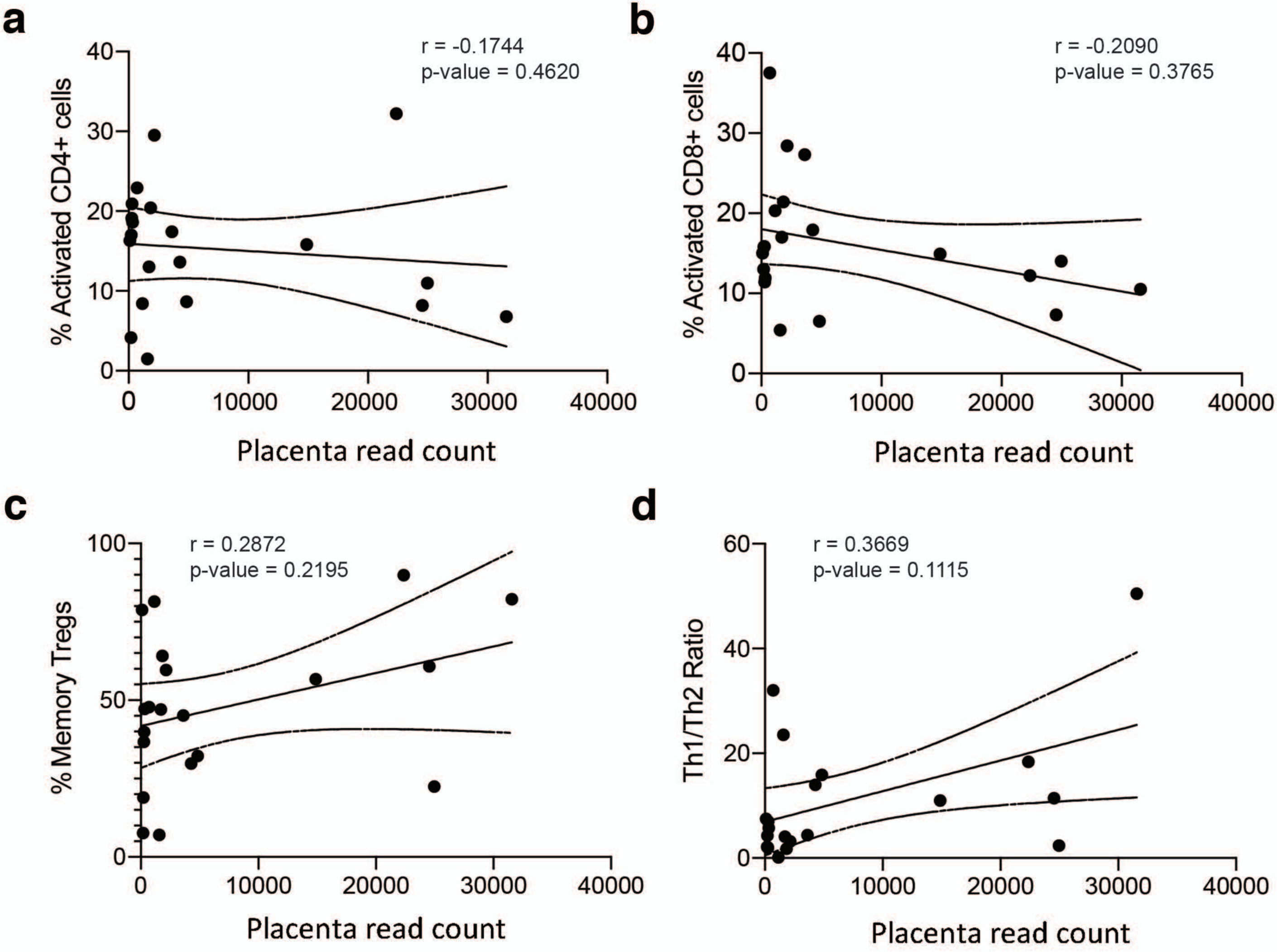

Supplemental Figure S13. Correlation between placental read counts and frequencies of activated CD4+ T cells (a), activated CD8+ T cells (b), memory Treg cells (c), and the Th1/Th2 ratio (d).

Sample #	Age	BMI	GA	Tissues	Question Answers
801	35	21.3	5.6	Placenta, decidua, decidual immune cells	Q1: No. Q2: No. Q3: No. Q4: No. Q5: No.
802	40	32.8	9.1	Placenta, decidua	Q1: No. Q2: No. Q3: No. Q4: No. Q5: No.
803	21	26	7.4	Placenta, decidua, decidual immune cells	Q1: No. Q2: No. Q3: No. Q4: No. Q5: No.
812	22	18.1	5.3	Placenta, decidua, decidual immune cells	Q1: No. Q2: No. Q3: No. Q4: No. Q5: No.
814	21	22.7	11	Placenta, decidua, fetal bowel	Q1: Yes. Q2: Yes. Q3: No. Q4: Yes. Q5: No.
815	21	20.1	8.5	Placenta, decidua, decidual immune cells	Q1: No. Q2: No. Q3: No. Q4: No. Q5: No.
816	40	22.6	9.3	Placenta, decidua, decidual immune cells	Q1: No. Q2: No. Q3: No. Q4: No. Q5: No.
817	26	21.5	6.6	Placenta, decidua, decidual immune cells	Q1: No. Q2: Yes. Q3: No. Q4: Yes. Q5: No.

818	39	28.3	13.6	Placenta, decidua, decidual immune cells, fetal bowel	Q1: No. Q2: No. Q3: No. Q4: No. Q5: No.
820	27	36.5	10	Placenta, decidua, decidual immune cells	Q1: No. Q2: No. Q3: No. Q4: No. Q5: No.
821	27	23.9	8.1	Placenta, decidua, decidual immune cells	Q1: No. Q2: Yes. Q3: No. Q4: Yes. Q5: No.
822	32	20.4	8.3	Placenta, decidua, decidual immune cells	Q1: No. Q2: No. Q3: No. Q4: No. Q5: No.
829	30	31.1	17.1	Placenta, Fetal gut, liver, spleen	Q1: No. Q2: Yes. Q3: No. Q4: No. Q5: No.
830	23	38.8	10	Placenta, decidua, decidual immune cells	Q1: No. Q2: No. Q3: No. Q4: No. Q5: No.
831	24	22.3	8.6	Placenta, decidua, decidual immune cells	Q1: No. Q2: Yes. Q3: No. Q4: No. Q5: No.
832	29	27.5	9.1	Placenta, decidua, decidual immune cells	Q1: No. Q2: No. Q3: No. Q4: Yes. Q5: No.

833	30	22.1	7.5	Placenta, decidua, decidual immune cells	Q1: No. Q2: No. Q3: No. Q4: No. Q5: No.
834	34	34.4	11.5	Placenta, decidua, decidual immune cells	Q1: No. Q2: Yes. Q3: No. Q4: No. Q5: No.
837	30	23	9.3	Placenta, decidua, decidual immune cells	Q1: No. Q2: No. Q3: No. Q4: No. Q5:No.
838	27	26.5	10	Placenta, decidua, decidual immune cells	Q1: No. Q2: No. Q3: No. Q4: No. Q5: No.
839	23	29.1	7.3	Placenta, decidua, decidual immune cells,	Q1: No. Q2: No. Q3: No. Q4: No. Q5: No.
841	21	22.7	14.4	Placenta, decidua, decidual immune cells, fetal gut	Q1: No. Q2: No. Q3: No. Q4: No. Q5: No.
843	19	30.8	12	Placenta, decidua, decidual immune cells	Q1: No. Q2: Yes. Q3: No. Q4: No. Q5: No.
846	39	33.3	9.6	Placenta, decidua, decidual immune cells	Q1: No. Q2: No. Q3: No. Q4: No. Q5: No.
847	27	22.6	9.5	Placenta, decidua, decidual immune cells	Q1: No. Q2: Yes. Q3: No. Q4: No. Q5: No.

Supplementary Table S1. Patient cohort demographics and answers to questions 1-5. Q1: “Did you take anti-hypertensive medications pre/post pregnancy?” Q2: “Did you smoke?” Q3: “Are you a diabetic/do you have diabetes?” Q4: “Do you take anti-inflammatory medications?”Q5: “Are you presently taking antibiotics?”.

Sample	Mean CT	Quantity	Undiluted Quantity
801P	23.82	2881	28806
801D	23.62	3250	32500
802P	22.63	6024	60242
802D	23.51	3492	34922
803P	23.63	3231	32306
803D	22.89	5107	51068
812P	22.64	5987	59866
812D	22.04	8656	86556
814P	24.13	2365	23651
814D	24.12	2382	23821
814G	21.75	10418	104184
815P	21.31	13653	136535
815D	21.68	10872	108720
816D	21.00	15000	15000
816P	20.00	25000	16000
817D	21.62	11246	112455
818P	21.24	14246	142464
818D	22.18	7962	79616
818G	18.53	76424	764236
820P	21.00	23000	5800
820D	22.00	12000	4200
821P	20.00	25000	8600
822P	22.64	5973	59725
822D	22.77	5532	55323
829P	23.01	4744	47435
829G	17.62	134640	1346397
829S	22.49	6582	65816
829L	18.24	91565	915650
830P	21.74	10485	104855
830D	23.14	4386	43858
831P	25.60	950	9497
831D	23.62	3252	32520
832P	24.09	2425	24251
832D	24.38	2028	20282
833P	22.58	6201	62005
833D	24.28	2165	21647
834P	22.61	60767	60767
834D	20.82	18560	185600
837P	22.52	6434	64342
837D	22.55	6304	63042
838P	26.96	409	4087

838D	27.44	304	3040
839P	23.36	3828	38276
839D	25.72	885	8846
841P	31.49	25	247
841D	24.17	2320	23202
841G	24.02	2544	25444
843P	24.23	2227	22266
846P	23.17	4315	43151
846D	24.99	1394	13938
847P	22.74	5613	56134
847D	21.09	15673	156732
830 PBS wash	29.34	94	936
820 PBS wash	32.57	13	126
834 PBS wash	32.80	11	109
839 PBS wash	32.86	10	105
844 PBS wash	33.30	8	80
837 PBS wash	33.07	9	93
841 PBS wash	32.22	16	157
843 PBS wash	32.83	11	107
831 PBS wash	32.90	10	103
816 PBS wash	32.67	12	118

Supplementary Table S2. Quantitative 16S PCR mean CT values and quantity by sample. P = placenta, D = decidua, G = gut (intestine), S = spleen, L = Liver, PBS = phosphate buffered saline.

Cell Marker	Fluorophore	Supplier	Reference Number	Clone	Dilution
CD45	PeCy5	eBioscience	15-0459-42	HI30	1:75
CD3	PerCP710	eBioscience	46-0036-42	SK7	1:75
CD8	APCH7	BD Biosciences	560179	SK1	1:30
CD4	Alexa700	BD Biosciences	557922	RPA-T4	1:50
CXCR3	APC	BD Biosciences	561732	1C6/CXCR3	1:25
CCR6	PeCy7	eBioscience	25-1969-42	R6H1	1:50
CD25	ef450	eBioscience	48-0259-42	BC96	1:25
CD45RO	PE	eBioscience	12-0457-42	UCHL1	1:50
HLA-DR	V500	BD Biosciences	561225	G46-6	1:50
Viability Dye	FVD520	eBioscience	65-0867-14	N/A	1:1000
FOXP3	PE-CF594	BD Biosciences	562421	259D/C7	1:25

Supplementary Table S3. Flow cytometry antibodies used in this study.
